# Key competencies for Korean nurses in prenatal genetic nursing: experiential genetic nursing knowledge, and ethics and law

**DOI:** 10.3352/jeehp.2020.17.36

**Published:** 2020-11-26

**Authors:** Gyeyoung Shin, Myunghee Jun, Hye-Kyung Kim, Michael Wreen, Sylvia Mimi Kubsch

**Affiliations:** 1 College of Nursing, Shinhan University, Dongducheon, Korea; 2 Department of Nursing and Health Studies, University of Wisconsin–Green Bay, Green Bay, WI, USA; 3 Department of Philosophy, University of Wisconsin–Green Bay, Green Bay, WI, USA; 4 Department of Philosophy, Marquette University, Milwaukee, WI, USA; Hallym University, Korea

**Keywords:** Genetics, Korea, Nursing education, Nursing ethics, Prenatal care

## Abstract

**Purpose:**

This study aims at determining the competencies of Korean nurses in prenatal genetic nursing.

**Methods:**

First, a 3-round Delphi survey was conducted to establish prenatal genetic nursing competencies. Second, a prenatal genetic nursing education program (PGNEP), incorporating the findings from the Delphi survey, was designed. Third, a single group pre- and post-quasi-experimental study at a PGNEP workshop was conducted to assess the effectiveness of the integration of the competencies into the PGNEP with the measurements of knowledge about prenatal genetic testing and nursing (K-PGTN) and information needs about prenatal genetic testing and nursing (I-PGTN). Finally, the identified competencies were reexamined for their clarity.

**Results:**

Based on the Delphi survey 78 competency components were identified. The components were then classified under 10 categories, which were organized under 4 domains. The domain of “experiential genetic nursing knowledge” and the domain of “ethics and law” were ranked as the first and the second in significance. The quasi-experimental study showed that the mean scores in K-PGTN were significantly increased from 8.19±2.67 to 11.25±2.51 (P<0.001). The mean scores of “ethics and law” in I-PGTN decreased significantly (P=0.023). The headings of 4 categories and 2 domains were revised.

**Conclusion:**

This study identified competencies for prenatal genetic nursing and nursing education in Korea. There is a need for nursing instructors and researchers to improve the competencies of nurses in the identified areas. Particular emphasis should be placed on experiential nursing knowledge and on ethics and law related to prenatal genetic nursing.

## Introduction

### Background/rationale

Regardless of the academic preparation, position, or clinical specialty of practicing nurses, their roles and duties have dramatically changed because of advances in genetics [1]. Nursing educators have responded to the advances in genetics by offering instruction in genetics and in the implications of genetics for clinical practice [2-5]. But questions regarding the comprehensiveness of genetic nursing education still remain [6,7]. In particular, a list of competencies for prenatal genetic nursing and education in Korea is not yet fully established. “Prenatal genetic nursing” refers to nursing care for pregnant women and their families, whose fetuses are at risk for genetic reasons. Such women have a greater need for prenatal genetic testing and diagnosis [8]. The point is not new; the need for establishing competencies in prenatal genetic nursing has been noted by many nursing researchers [8-10].

Prenatal genetic technologies have been rapidly integrated into prenatal health care practice. Non-invasive prenatal testing (NIPT) and preimplantation genetic diagnosis are now relatively common clinical procedures. From an ethical point of view, particular concerns include the termination of pregnancy, autonomous decision making, and respect for life. The development of prenatal genetic technology, however, has created a particular kind of concern in Korea. Recent demographic changes in Korea have necessitated comprehensive prenatal genetic nursing for many pregnant women. The reason is evident and the situation a little short of urgent. In Korea in 2016, the median age of first-time mothers (the women who gave birth for the first time) was 31.6 and the median age of the women who gave birth was 32.6. This is the highest among Organization for Economic Co-operation Development countries [11]. Almost 1 in 3 women who give birth in Korea are now aged 35 or over. Due to high-risk age-related factors, the need for these women for professional genetics-related medical services is especially acute. Unfortunately, the problem has not been sufficiently addressed; even the services provided by certified prenatal genetic counselors do not exist. In this difficult situation, nurses caring for high-risk pregnant women have played, and have had to play, a significant role. This role has sometimes included prenatal genetic counseling. However, current nursing education does not sufficiently prepare Korean nurses for the full range of competencies needed for effective prenatal genetic nursing [10]. This means, first, that essential competencies for the professional prenatal genetic nursing must be identified. Second, nursing education for nurses in the area of prenatal genetic nursing must respond accordingly. Therefore, it is necessary to develop educational strategies and to provide programs so that nurses are able to meet the health care needs of high-risk pregnant women.

### Objectives

The study aimed to determine competencies for Korean nurses needed in prenatal genetic nursing and nursing education. Specifically, it aimed to identify the key area of competencies in order to facilitate more efficient education for nurses. The findings from the study can help to improve Korean prenatal genetic nursing and education. The results will be a good support to add prenatal genetic nursing competency to the continuous professional nursing education.

## Methods

### Ethics statement

The proposal for the project was approved by the ethical review board at the Daejeon University, Korea (IRB approval no., Djomc-87). Informed consent was obtained from all of the participants in the Delphi survey and quasi-experimental study.

### Study design

To establish a preliminary list of competencies, a 3-round Delphi survey was conducted. A single group pre- and post-test quasi-experimental study was conducted to measure the effectiveness of the prenatal genetic nursing education program (PGNEP). Measuring the effectiveness enabled the validation of the findings of the Delphi survey.

### Participants

Twenty medical and nursing professionals participated in the Delphi survey: 8 physicians (40.0%), 7 nursing faculty (35.0%), 4 clinical nurses (20.0%), and 1 genetic counselor (5.0%). Physicians and nursing faculty comprise 75% of the participants. Physicians who treat patients with possible prenatal genetic problems are most knowledgeable about services that need to be provided to such patients. On the other hand, nursing faculty know which competencies nurses need to master in the area. The mean age of participants in the Delphis survey was 42.00±10.15. Thirty-two nurses participated in the quasi-experimental study. The average age was 43.91±10.37. Eighteen participants (56.3%) held a doctoral degree and 7 (21.9%) held a bachelor or master’s degree in nursing. Thirteen participants (40.6%) were working at a hospital and 19 (59.4%) were nursing educators (Table 1).

### Setting

**Fig. 1.**
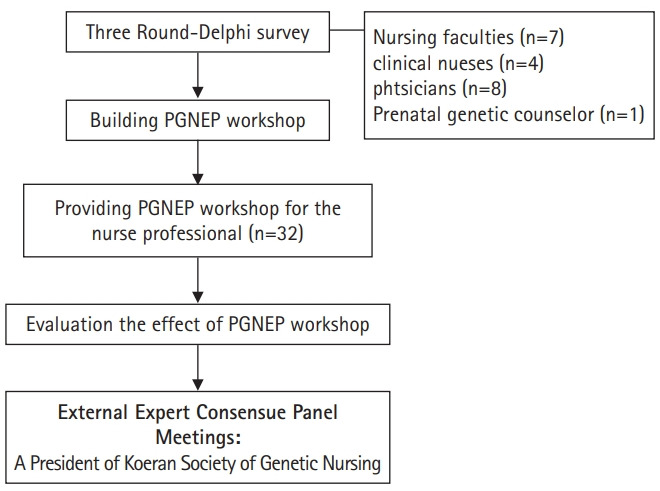
Flowchart of the study process. PGNEP, prenatal genetic nursing educational program.

Diagram of the study process was presented in Fig. 1. The study had 3 stages. The first comprised a 3-stage Delphi survey, a classification of the findings, and the design of a PGNEP. The second comprised a quasi-experimental study. Finally there was a consultation with an external expert, and the exploration of its implications.

The first component of the first stage was a 3-round Delphi survey. It was conducted from June 2012 to July 2013. In the first round of the Delphi survey 5 open questions were asked in relation to prenatal genetic nursing and education (Supplement 1). Its English version is also provided for readers (Supplement 2). The questions concern competency components. The competencies themselves concern prenatal genetic education and counseling, neonatal anomalies, prenatal genetic testing, and curricula for prenatal genetic education. Based on the qualitative analysis, 78 components of competencies were identified (Supplement 3). The 78 competencies were then classified under 10 categories. The 10 categories were identified on the basis of similarities between and overlap of competencies. For further clarity and ease of comprehension, the 10 categories were grouped under 4 domains: basic genetic knowledge; ethics and law; experiential genetic nursing knowledge; and prenatal genetic testing knowledge (Table 2).

Two more surveys were conducted to further validate the list of competencies and to establish the relative rankings among the 10 categories. Subsequent to the third round of the Delphi survey, a framework for the quasi-experimental study was developed and a PGNEP workshop was designed in May 2013. The design was the result of a collaborative effort, with the first and the second authors receiving advice and feedback from 2 external experts: a medical professor at the department of obstetrics and gynecology of Seoul National University, and the President of Korean Society of Genetic Nursing in 2013. During the designing stage of the workshop, a new prenatal genetic test, cell-free fetal DNA or NIPT, was introduced into Korean prenatal genetic nursing practice. Instruction on it was included in the PGNEP workshop [9].

At the second stage, a quasi-experimental study at a PGNEP workshop was conducted from August 19 through August 21, 2013 on Chung-Ang University campus in Korea. The 32 participants were recruited through announcements on the homepages of major Korean nursing academic societies and the Korean Nurse Association. A 3-day PGNEP workshop was run and a quasi-experimental study conducted. Thirty-two nurses participated in the workshop and the quasi-experimental study. Three-day prenatal genetic nursing educational program (PNGEP) syllabus is presented in Supplement 4.

Finally, for additional validation of the outcomes of the 2 studies, one of the external experts, the President of Korean Society of Genetic Nursing, vetted the relevance of the 10 categories and 4 domains. With her feedback, refinement of the competency components, the categories, and the domains was affected. This occurred from January 2015 through December 2018.

### Measures

The quasi-experimental study served 2 purposes. The first was to assess changes in competency levels after the PGNEP workshop. Such assessment was needed to evaluate the effectiveness of the integration of the competencies into the PGNEP. The second purpose was to determine participants’ satisfaction with the framework of the PGNEP. Changes in competency levels were assessed in 2 areas: “knowledge about prenatal genetic testing and nursing (K-PGTN)” and “information needs about prenatal genetic testing and nursing (I-PGTN)”. These 2 measurement tools were based on previous research on the knowledge and information needs of nurses and pregnant women about prenatal genetic screening and diagnosis [10,12]. However, the measurement tools of the previous study were modified for the study at hand (Supplement 5). Supplement 6 is the English version for readers. There was permission of the use of tool for K-PGTN by original author. The competency level in K-PGTN was measured by the “yes” or “no” answers to the 15 test items. The higher the score, the greater the knowledge of prenatal genetic testing and nursing. Cronbach’s α was 0.59–0.67.

The I-PGTN was measured by 21 test items. This tool was used under the Creative Commons license CC-BY. The items fell within 4 domains: The domain of basic genetic knowledge included 3 items; the domain of ethics and law 3 items; the domain of experiential genetic nursing knowledge 4 items; the domain of prenatal genetic testing knowledge 11 items. The participants responded on a 5-point Likert scale. Higher scores indicated greater information needs for an item. Cronbach’s α was 0.96–0.99.

Participant satisfaction level with the program framework and content was measured on 5 of the 9 items on a 5-point Likert scale, and on 3 of the open questions. The measurment tool for the participant satisfaction was crafted by the first and second authors. Higher scores indicate greater satisfaction with program content and framework.

Finally, the identified competency components, categories, and domains were refined by the researchers and confirmed by an external genetic nursing expert. Four researchers (G.S., M.J., H.K.K., and M.W.) vetted the conceptual clarity of each component, the validity of grouping components into categories and domains, and the relevance of their headings.

### Sample size

According to the posthoc power analysis for paired t-test based on given effect size 0.5, alpha error probability 0.05, and total sample size 32, power was 0.869 (G*Power ver. 3.1494; Heinrich-Heine-Universität Düsseldorf, Düsseldorf, Germany; http://www.gpower.hhu.de/) [13].

### Statistical methods

A qualitative content analysis of the outcomes of the first round of the Delphi survey was conducted. Descriptive statistics were used to measure the outcomes of the second and third rounds of Delphi survey, while only a paired t-test was used to measure the outcomes of the quasi-experimental study. The outcomes of descriptive statistics and paired t-test were analyzed with IBM SPSS Statistics ver. 27.0 (IBM Corp., Armonk, NY, USA).

## Results

### Delphi survey

The study resulted in the identification of 78 competency components (Supplement 3), 10 categories of classification, and 4 broader domains of interest. Of the 10 categories, “‘basic (molecular) genetic knowledge,” “general knowledge related to genetic testing,” “knowledge related to anomalies,” and “knowledge related to conception and pregnancy” fell under the domain of basic genetic knowledge. “Ethical, legal, and social issues” (ELSIs) and “social welfare” fell under the domain of ethics and law. “Clinical genetic nursing knowledge” and “knowledge from case study and practicum” fell under the domain of experiential genetic nursing knowledge. “Prenatal genetic testing” and “tests for genetic diseases” fell under the domain of prenatal genetic testing knowledge. The 10 categories were then ranked on the basis of the scores participants gave to individual competency components. “Clinical genetic nursing knowledge” was at the top of the list. “ELSIs” was second (Table 2). These 2 categories were more heavily weighted in program design (Supplment 4).

The PGNEP was designed on the basis of the 10 categories, with an emphasis on the 3 top-ranked categories. The knowledge category and the value category thus were the foci for the 3-day workshop (Supplemnet 4).

### Quasi-experimental study

After participants completed the PGNEP workshop, the mean score of knowledge (K-PGTN) significantly increased from 8.19±2.67 to 11.25±2.51 (P<0.05) (Table 3). The mean score of total information need (I-PGTN) decreased from 95.00±8.86 to 91.53±15.16. But this change was not statistically significant (P>0.05) except 1 subscale; the information need for ethics and law (domain 2) decreased significantly (P=0.023) (Table 3). The mean score of the program content satisfaction was 22.51±1.73 (90.0%). Raw data of responses are available from Dataset 1.

Finally, the identified competency components, categories, and domains were refined by 4 researchers (G.S., M.J., H.K.K., and M.W.) and confirmed by an external genetic nursing expert. Refinement resulted in changed headings for 4 categores within the domains of experiential genetic nursing knowledge and ethics and law, as well as the present headings of expeiential genetic nursing knowledge and ethics and law. The final validations came from the President of Korean Society of Genetic Nursing in 2013.

## Discussion

### Interpretation

The Delphi survey was conducted with prenatal genetic nursing experts. The outcomes of the study establish a list of 78 competencies for prenatal genetic nursing.

First, Korean nursing educators and nurses have known that nurses in prenatal genetic nursing should be better educated; advances in genetic technologies alone long ago made that evident. But the need for more, new, and better education is all the more urgent because of the dramatic shift in the age at which Korean women are now giving birth [9,10,14]. In Korea, prenatal genetic counselors are lacking, and nurses must fill in the gap [12,14]. The Delphi survey is an attempt to articulate the competencies required to fill in the gap for the unique circumstances of prenatal genetic nursing in Korea.

Second, the Delphi survey identifies the most significant domains for competencies as those of knowledge and value. “Experiential genetic nursing knowledge” and “ethics and law” as the 2 most vital domains. “Experiential genetic nursing knowledge” includes 2 sub-categories—knowledge from case study and practicum and clinical genetic nursing knowledge, which are necessary for nurses’ communication with patients and their families and medical and emotional support for them (Supplement 3). In other words, “experiential genetic nursing knowledge” is mainly concerned with knowing experientially how to communicate with, educate, and counsel pregnant women. This implies that clinical genetic nursing knowledge needs to be acquired through case studies and practicums [15].

Information needs for all categories decreased, but not all of them significantly; the information need for “ethics and law” did decrease significantly. This latter outcome implies that the need for “ethics and law” was largely satisfied by the program. The shortness of the workshop may explain why the information needs for other domains did not decrease significantly.

One subscale, “ethics and law”, should be understood in terms of value, as opposed to factual knowledge, whether technical or not. At base, value is focused on good and bad, right and wrong, legal and illegal. But factual knowledge and value knowledge are intertwined in all healthcare practice, and value itself is idle without knowledge of value. Value-knowledge is central to prenatal genetic nursing. It includes knowledge of ELSIs related to prenatal genetics.

The study demonstrates the need for value competency in prenatal genetic nursing. That means that, nurses need values education as well as experiential genetic nursing knowledge. They need but currently lack the capacity to reason, in a responsible, effective, and value-sensitive way, on prenatal genetic ethical issues. Such a capacity is requisite for the capacity to support patient decision-making.

The case at hand is particularly pointed in the regard. In Korea, termination of pregnancy, due to fetal anomaly, is legally prohibited by the Mother and Child Health Law. A patient’s decision to terminate implies illegal activity, which may harm the patient herself and the fetus. But the decision to terminate is never simple. A patient may be in a unique personal, cultural, or religious situation which may lead to illegal termination [9,10,12].

Third, a framework for a PNGEP is in place. Four domains for structuring a PGNEP, with a special emphasis on the domains of “experiential genetic nursing knowledge” and “ethics and law”, have been developed.

The quasi-experimental study thus supported the validity of the list of competencies that had been identified. It was the participants’ satisfaction with the PGNEP that provided such support. The participants appreciated the value of experiential genetic knowledge and the importance of nurse’ values and ethical decision-making abilities. Such satisfaction also affirms the effectiveness and value of the PGNEP itself. The PGNEP provided at least some needed knowledge of prenatal genetic screening and diagnostic testing. The categories and domains of competencies determined the framework and content of the workshop.

### Limitation

This study has potential limitations. First, the measurement tools used were drawn from previous research. They might not be perfect for the specific research pursued here. Ideal would be to develop new tools specifically for the subject area. However, previous research was in a similar subject area, and the measurement tools used were certainly relevant and yielded significant results. Second, the competency components, categories, and domains may change in response to new technology and changing social and political circumstances. The competency components identified are the products of Korea’s unique cultural, social, political, and legal circumstances. Caution is thus needed in generalizing the study’s findings and extending them to other communities.

### Conclusion

A list of 78 competencies for prenatal genetic nursing and a framework for effective PGNEP have been identified. The framework focuses on experiential genetic nursing knowledge and value education. A PGNEP which integrated such competencies is shown to be effective and valuable. Nursing educators should include instruction in these competencies into nursing curriculums. But it is also vital for nursing researchers to refine the list of competencies identified and to build upon the framework of the PGNEP developed. Findings of this study will be able to provide the supporting evidence to include the prenatal genetic nursing compentency to the continuous professional nursing education.

## Figures and Tables

**Table 1.** General characteristics of study participants

Category	Type	Delphi survey (N=20)	PGNEP workshop (N=32)
Education level	Bachelor	4 (20.0)	7 (21.9)
	Master	-	7 (21.9)
	PhD	16 (80.0)	18 (56.3)
Job title	Physician	8 (40.0)	-
	Clinical nurse	4 (20.0)	13 (40.6)
	Nursing faculty	7 (35.0)	19 (59.4)
	Genetic counselor	1 (5.0)	-
Current working place	Hospital	12 (60.0)	13 (40.6)
	Education	8 (40.0)	19 (59.4)

Values are presented as number of participants (%). PGNEP, prenatal genetic nursing education program.

**Table 2.** Delphi survey result for developing prenatal educational program for Korean clinical nurses (total=78)

Domain	Category (no. of elements)	Rank
Basic genetic knowledge	1. Basic (molecular) genetic knowledge (n=3)	6
	2. General knowledge related to genetic test (n=14)	8
	3. Knowledge related to aAnomalies (n=6)	5
	4. Knowledge related to conception and pregnancy (n=5)	10
Ethics and law	5. Ethical, legal, social issues (n=4)	2
	6. Social welfare (n=3)	7
Experiential genetic nursing knowledge	7. Clinical genetic nursing knowledge (n=11)	3
	8. Knowledge from case study and practicum (n=2)	1
Prenatal genetic testing knowledge	9. Prenatal genetic testing (n=12)	4
	10. Tests for genetic disease (n=18)	9

**Table 3.** Effect of prenatal genetic nursing education on the knowledge and information need (N=32)

Variable	Pre-test	Post-test	Difference (post–pretest)	t-value	P-value
Knowledge (K-PGTN) (n=15)	8.19±2.67	11.25±2.51	3.06±2.96	5.85	<0.001
Information need (I-PGTN)					
I1 (n=3)	13.31±1.62	12.84±2.49	-0.47±2.46	-1.08	0.290
I2 (n=3)	14.13±1.21	13.31±2.01	-0.81±1.93	-2.39	0.023
I3 (n=4)	17.88±2.14	17.53±2.74	-0.34±3.02	-0.64	0.525
I4 (n=11)	49.69±4.78	47.84±8.46	-1.84±8.01	-1.3	0.202
Total (n=21)	95.00±8.86	91.53±15.16	-3.47±14.44	-1.36	0.184

Values are presented as mean±standard deviation, unless otherwise stated. K-PGTN, knowledge about prenatal genetic testing and nursing; I-PGTN, information need about prenatal genetic testing and nursing; I1, information need for genetic disease; I2, information need for ethics and law; I3, information need for experiential genetic nursing knowledge; I4, information need for prenatal genetic testing.

## Footnotes

**Data availability** Data files are available from Harvard Dataverse: https://doi.org/10.7910/DVN/TNAICP Dataset 1. Raw data of responses from 32 participants in quasi-experimental study.

## Supplementary materials

Data files are available from Harvard Dataverse: https://doi.org/10.7910/DVN/TNAICP

Supplement 1. Delphi survey tool (Korean version).

Supplement 2. Delphi survey tool (English version).

Supplement 3. Delphi survey result on the essential category and competency components for the prenatal genetic nursing care and education in South Korea.

Supplement 4. Three-day prenatal genetic nursing educational program syllabus.

Supplement 5. The questionnaire of the quasi-experimental study (Korean version).

Supplement 6. The questionnaire of the quasi-experimental study (English version).

Supplement 7. Audio recording of the abstract.
