# Supplementary material for: Key competencies for Korean nurses in prenatal genetic nursing: experiential genetic nursing knowledge, and ethics and law
Source: J Educ Eval Health Prof. 2020 Nov 26;17:36. doi: 10.3352/jeehp.2020.17.36 (PMC7847985; doi:10.3352/jeehp.2020.17.36)
Supplement: Supplementary file 3 — Supplement 2. Delphi survey tool (English version). [file jeehp-17-36-suppl2.docx]

**Supplement 2.** Delphi survey tool (English version)

**Delphi survey for developing a prenatal genetic nursing and educational program in South Korea**

| The purpose of this Delphi survey is to develop a prenatal genetic nursing and educational program for Korean nurses. You will be asked 3 times to respond to this survey.  All the information collected from this questionnaire is private and will be processed anonymously. Participation in this study is voluntary. You may choose to take part in the study, not to take part in this study, or decide to take part in the study and later change your mind later and withdraw. The information you provide will be kept strictly confidential. If you have any questions concerning the research, please contact the primary investigator, Dr. Myunghee Jun (jun7710@dju.ac.kr).  Thank you for your participation in this survey. |
| --- |

**Question: first session**

1. What genetic tests are administered at a prenatal clinic? Please write 5 tests which you think are important.

1)

2)

3)

4)

5)

2. What should nurses inform pregnant women about as far as prenatal genetic tests are concerned? Please indicate everything you think important.

1)

2)

3)

4)

5)

3. What should prenatal clinic nurses know about neonatal anomalies? Please indicate everything you think important.

1)

2)

3)

4)

5)

4. What should prenatal clinic nurses know about prenatal genetic tests? Please indicate everything you think important.

1)

2)

3)

4)

5)

5. What should an educational curriculum for prenatal clinic nursing include? Please indicate everything you think important.

1)

2)

3)

4)

5)

6. Please write down anything else you think important as far as education in prenatal genetic nursing is concerned.

※ Please indicate the following information about yourself (this is for demographic purposes only):

**1. Job**

① Doctor/physician ( ) ② Clinical nurses ( )

③ Nursing faculty ( ) ④ Genetic counselor ( ) ⑤ Other (specify: )

**2. Gender**

① Male ( ) ② Female ( )

**3. Age: ( ) year**

**4. Education**

① Bachelor ( ) ② Master ( ) ③ PhD ( )

**5. Clinical career**

__________year __________month

**6. Do you currently administer prenatal genetic tests?**

① Yes ( ) ② No ( )

**7. Have you currently certified genetic counselor?**

① Yes ( )

② No ( )
