# Supplementary material for: Key competencies for Korean nurses in prenatal genetic nursing: experiential genetic nursing knowledge, and ethics and law
Source: J Educ Eval Health Prof. 2020 Nov 26;17:36. doi: 10.3352/jeehp.2020.17.36 (PMC7847985; doi:10.3352/jeehp.2020.17.36)
Supplement: Supplementary file 4 — Supplement 3. Delphi survey result on the essential category and competency components for the prenatal genetic nursing care and education in South Korea. [file jeehp-17-36-suppl3.docx]

**Supplement 3.** Delphi survey result on the essential category and competency components for the prenatal genetic nursing care and education in South Korea

| Category (no. of items) | Competency components | 3rd round mean±SD |
| --- | --- | --- |
| 1. Basic (molecular) genetic knowledge (3) | DNA (genetic) variation/mutation | 4.20±0.70 |
|  | Molecular genetics (cell division, chromosomal structure, and function) | 4.15±0.67 |
|  | Inheritance pattern | 3.80±0.95 |
| 2. General knowledge related to genetic test (14) | Basic knowledge related to prenatal genetic tests (type, goal, method, gestational period of taking test, interpretation of the test results, complication, nursing care before and after test) | 4.90±0.31 |
|  | Prenatal genetic test according to gestational period | 4.90±0.31 |
|  | Prenatal genetic test specific to genetic disease | 4.30±0.73 |
|  | Limitation of the genetic test | 4.10±0.55 |
|  | Proper time to inform the test results | 4.10±0.64 |
|  | Management the genetic fetal disease before birth | 4.05±0.69 |
|  | Expenses and insurance related to genetic test | 4.00±0.65 |
|  | Consideration of the prenatal test affecting to the fetus | 3.95±0.83 |
|  | Management the genetic disease after birth | 3.90±0.85 |
|  | Recent prenatal genetic test (e.g., preimplantation testing, preimplantation genetic diagnosis, harmony prenatal test) | 3.70±0.66 |
|  | Sonographic knowledge related to prenatal test | 3.5±0.76 |
|  | Reliability and validity of the molecular genetic test | 3.10±1.17 |
|  | Molecular analysis method of the genetic test | 2.85±1.04 |
|  | Criteria of the reliable laboratory for genetic test | 2.68±1.11 |
| 3. Knowledge related to Anomalies (6) | Congenital anomalies which occur frequently | 4.40±0.60 |
|  | Management and prognosis of congenital anomalies | 4.20±0.70 |
|  | Fetal anomalies related to drug exposure or infection | 4.10±0.64 |
|  | Congenital anomalies whose prenatal diagnoses are possible | 4.00±0.75 |
|  | Understanding of the genetic disease | 3.95±0.85 |
|  | Mendelian/non-Mendelian genetic diseases | 3.80±1.01 |
| 4. Knowledge related to conception and pregnancy (5) | Conception and pregnancy | 3.75±0.64 |
|  | Pathophysiology of pregnancy and delivery | 3.85±0.67 |
|  | Fetal health assessment | 3.70±0.73 |
|  | Sign and symptom according to the gestational period | 3.60±0.82 |
|  | Understanding personal meaning about their pregnancy | 3.35±0.88 |
| 5. ELSI (4) | Legal and social issues related to prenatal genetic test | 4.20±0.77 |
|  | Support patent’s decision making whether to keep or terminate the pregnancy | 4.45±0.51 |
|  | Bioethics | 4.45±0.51 |
|  | Current legal regulations related to women and children’s health and bioethics | 4.40±0.60 |
| 6. Social welfare (3) | Information about the available diagnostic genetic counseling centers | 4.10±0.64 |
|  | Connection to available social worker or supporting resource | 3.90±0.79 |
|  | Collaboration with other disciplines | 3.85±0.59 |
| 7. Clinical genetic nursing knowledge (11) | Types and methods of the prenatal genetic tests for nursing education | 4.85±0.37 |
|  | Knowledge for the nurses who perform prenatal genetic educating the pregnant women | 4.80±0.41 |
|  | Educational methods for prenatal genetic education for pregnant women | 4.60±0.60 |
|  | Counseling attitude: protecting privacy, providing accurate information | 4.50±0.61 |
|  | Communication skill | 4.45±0.76 |
|  | Psychological support | 4.35±0.81 |
|  | Educating pregnant women taking genetic test | 4.30±0.80 |
|  | Constructing and interpretation of pedigree | 4.20±0.89 |
|  | Risk calculation and assessment about the genetic disease | 4.15±0.81 |
|  | Family dynamics and communication among family members | 4.10±0.85 |
|  | Cultural, and religious consideration | 3.65±0.89 |
| 8. Knowledge from case study and practicum (2) | Case study | 4.40±0.60 |
|  | Clinical practicum | 4.40±0.68 |
| 9. Prenatal genetic testing (12–>13) | Quad/triple test (alpha-fetoprotein, estriol, human chorionic gonadotropin, inhibin-A) | 4.85±0.37 |
|  | Amniocentesis | 4.95±0.22 |
|  | CVS | 4.40±0.75 |
|  | Cordocentesis | 4.00±0.73 |
|  | Sonography (screening/detail) | 4.55±0.69 |
|  | Nuchal translucency measurement | 4.55±0.51 |
|  | Integral test | 4.35±0.59 |
|  | Chromosome analysis/full karyotyping/QF-PCR (21, 18, 16, X, Y) | 4.25±0.79 |
|  | Fetal heart electronic monitoring | 3.95±0.94 |
|  | Chromosomal analysis of the placenta after miscarriage | 3.74±0.93 |
|  | Chromosome microarray | 3.65±0.93 |
|  | Confirm the contamination of the sample from the CVS, amniocentesis, and umbilical cord | 3.25±0.85 |
| 10. Tests for genetic disease (18) | Down syndrome | 4.85±0.37 |
|  | Turner syndrome | 4.55±0.60 |
|  | Edward syndrome | 4.50±0.51 |
|  | Klinefelter syndrome | 4.40±0.68 |
|  | Patau syndrome | 4.25±0.64 |
|  | Hemophilia | 3.80±0.89 |
|  | CATH-22 (DiGeorge’s syndrome) | 3.70±0.80 |
|  | Duchenne muscular dystrophy | 3.50±0.76 |
|  | Fragile X | 3.50±0.89 |
|  | Skeletal dysplasia | 3.45±0.83 |
|  | Achondroplasia | 3.45±0.83 |
|  | Genetic test before implantation (preimplantation genetic test) | 3.35±1.14 |
|  | Myotonic dystrophy/myotonic muscular dystrophy | 3.35±0.75 |
|  | Hemoglobinopathies | 3.30±0.98 |
|  | Spinal muscular atrophy | 3.30±0.86 |
|  | Cystic fibrosis | 3.25±1.21 |
|  | Spinal muscular dystrophy | 3.25±0.97 |
|  | Hunter disease | 3.25±0.91 |

SD, standard deviation; ELSI, ethical, legal, and social issues; CVS, Chorionic villus sampling; QF-PCR, Quantitative fluorescent polymerase chain reaction.
