# Supplementary material for: Key competencies for Korean nurses in prenatal genetic nursing: experiential genetic nursing knowledge, and ethics and law
Source: J Educ Eval Health Prof. 2020 Nov 26;17:36. doi: 10.3352/jeehp.2020.17.36 (PMC7847985; doi:10.3352/jeehp.2020.17.36)
Supplement: Supplementary file 5 — Supplement 4. Three-day prenatal genetic nursing educational program syllabus. [file jeehp-17-36-suppl4.docx]

**Supplement 4.** Three-day prenatal genetic nursing educational program syllabus

| Date | Title | Lecturer (specialty) | Category^a)^ |
| --- | --- | --- | --- |
| Day 1 | Basic genetic knowledge related to prenatal care | Medical professor (molecular biology) | 1, 2 |
|  | Current genetic nursing issues | President of Korean Society of Genetic Nursing | 7, 8 |
|  | Genetic informatics and international genetic nursing trends | Professor in nursing informatics | 7 |
|  | Making pedigree and exercise | Oncology nurse practitioner | 5, 9 |
| Day 2 | Patterns of inheritance (AD, AR, XR, etc.) | Medical professor (pediatrics) | 1, 3 |
|  | Chromosomal disease and prenatal care  Inborn error of metabolism | Medical professor (pediatrics) | 3, 4 |
|  | High risk pregnant women’s experience | Nursing professor (genetic counseling) | 6, 7, 8 |
|  | Nurses’ role in the prenatal genetic screening and diagnostic testing | Nursing professor (maternal nursing) | 6, 7, 8, 9, 10 |
|  | Prenatal genetic screening and testing  Genetic counseling | Medical professor (perinatal obstetrics and gynecology) | 6, 7, 9, 10 |
|  | Prenatal diagnosis: multidisciplinary team approach and social welfare | Medical professor (congenital pediatric disease) | 6, 8 |
| Day3 | Single gene disorder | Medical professor (diagnostic laboratory medicine) | 3 |
|  | Genetic testing technology | Medical professor (diagnostic laboratory medicine) | 9, 10 |
|  | Genetic history taking and assessment | Oncology nurse practitioner (genetic nursing) | 5 |
|  | Prenatal genetic diagnosis and legislative issue | Medical doctor (OBGY & law) | 5, 6 |
|  | ELSI | Nursing professor (oncology, genetic nursing) | 5 |
|  | Resources for patients and family | Nursing professor (genetic nursing) | 6 |

AD, autosomal dominant; AR, autosomal recessive; XR, X-linked recessive; OBGY, obstetrics and gynecology; ELSI, ethical, legal, and social issues.

^a)^Domains established by the Delphi Survey result in this study (See Table 2 and Supplement 1).
