# Supplementary material for: Key competencies for Korean nurses in prenatal genetic nursing: experiential genetic nursing knowledge, and ethics and law
Source: J Educ Eval Health Prof. 2020 Nov 26;17:36. doi: 10.3352/jeehp.2020.17.36 (PMC7847985; doi:10.3352/jeehp.2020.17.36)
Supplement: Supplementary file 7 — Supplement 6. The questionnaire of the quasi-experimental study (English version). [file jeehp-17-36-suppl6.docx]

**Supplement 6.** Measurement tools for the quasi experiment (English version)

**Knowledge about prenatal genetic testing and nursing**

| Items | Yes | No |
| --- | --- | --- |
| B-3. Prenatal genetic screening and diagnostic testing are to determine whether a fetus has Down syndrome. |  |  |
| B-4. Prenatal genetic screening and diagnostic testing refer to amniocentesis. |  |  |
| B-5. A nuchal translucency scanning test is performed to detect neural tube defects of the fetus during pregnancy. |  |  |
| B-7. If the thickness of a pregnant woman’s nuchal translucency scanning is measured at more than 1.0 mm, she should undergo amniocentesis. |  |  |
| B-8. A cffDNA test is a genetic diagnostic test recommended following amniocentesis. |  |  |
| B-9. The family as well as the parents of fetus have the right to know prenatal genetic testing results and related information about the pregnancy. |  |  |
| B-10. In Korea, terminating a pregnancy before 24 gestational weeks is legally allowed if a fetal anomaly is detected. |  |  |
| C-1. All hereditary/genetic diseases of the fetus can be detected by prenatal genetic testing. |  |  |
| C-2. Hereditary and genetic diseases are easily discovered within one year after birth. |  |  |
| C-3. Common hereditary diseases are always transmitted to and expressed in the next generation. |  |  |
| C-4. If one parent is affected by an autosomal dominant disorder, there is a 25% chance that each child of that will inherit the same mutated gene. |  |  |
| E-1. All genetic diseases are inherited in accordance with the Mendelian principle of inheritance. |  |  |
| E-4. The chance that a male child and a female child will suffer from an autosomal dominant disease passed down from their parents is equal. |  |  |
| E-10. Humans have a total of 46 autosomes. |  |  |
| E-11. In addition to non-invasive prenatal testing, an ultrasound can identify a baby’s sex after 7 gestational weeks. |  |  |

**Information needs about prenatal genetic testing and nursing**

| Domain | Content | Strongly want to know | Want to know | No preference | Don’t want to know | Not at all |
| --- | --- | --- | --- | --- | --- | --- |
| Maternal serum screening test purpose |  |  |  |  |  |  |
| 4 | D1. First trimester screening test |  |  |  |  |  |
| 4 | D2. Second trimester screening test |  |  |  |  |  |
| 4 | D3. Integrated test |  |  |  |  |  |
| Amniocentesis |  |  |  |  |  |  |
| 4 | D4. Indication |  |  |  |  |  |
| 4 | D5. Side effect during amniocentesis |  |  |  |  |  |
| 4 | D6. Testing methods |  |  |  |  |  |
| 3 | D7. Care after amniocentesis |  |  |  |  |  |
| 4 | D8. Complication after amniocentesis |  |  |  |  |  |
| 3 | D9. Expenses |  |  |  |  |  |
| 3 | D10. Interpretation of the test results |  |  |  |  |  |
| 2 | D11. Management of the positive test results |  |  |  |  |  |
| 3 | D12. Management of the negative test results |  |  |  |  |  |
| Level II ultrasonography |  |  |  |  |  |  |
| 4 | D13. Purpose |  |  |  |  |  |
| 4 | D14. Interpretation of the test results |  |  |  |  |  |
| Genetic disease |  |  |  |  |  |  |
| 1 | D15. Downs syndrome |  |  |  |  |  |
| 1 | D16. Patau syndrome |  |  |  |  |  |
| 1 | D17. Edward syndrome |  |  |  |  |  |
| Chorionic villus sampling |  |  |  |  |  |  |
| 4 | D18. Purpose |  |  |  |  |  |
| 4 | D19. Interpretation of the test results |  |  |  |  |  |
| NIPT |  |  |  |  |  |  |
| 2 | D20. Understanding of NIPT |  |  |  |  |  |
| 2 | D21. Ethical considerations of NIPT |  |  |  |  |  |

NIPT, non-invasive prenatal testing.

**Satisfaction and evaluation of workshop**

1. **Workshop activity**

L1**.** Was this workshop effective in providing an overview of key prenatal genetic nursing knowledge and nursing care? ( )

1) Strongly agree 2) Agree 3) Neither disagree nor agree

4) Disagree 5) Strongly disagree

L2. Was the duration of the workshop (3 days) sufficient to meet the goals of the program? ( )

1) Strongly agree 2) Agree 3) Neither disagree nor agree

4) Disagree 5) Strongly disagree

L3. Did the workshop meet your expectation? ( )

1) Strongly agree 2) Agree 3) Neither disagree nor agree

4) Disagree 5) Strongly disagree

L4. Were the contents of the workshop and the expertise of lecturers helpful? ( )

1) Strongly agree 2) Agree 3) Neither disagree nor agree

4) Disagree 5) Strongly disagree

L5. Was the level of this workshop activities appropriate? ( )

1) Strongly agree 2) Agree 3) Neither disagree nor agree

4) Disagree 5) Strongly disagree

**2. Administrative aspects of the workshop**

|  | Strongly satisfied | Satisfied | Neither satisfied nor dissatisfied | dissatisfied | Strong dissatisfied |
| --- | --- | --- | --- | --- | --- |
| S1. Lunch |  |  |  |  |  |
| S2. Refreshment |  |  |  |  |  |
| S3. Registration process |  |  |  |  |  |
| S4. Facilities |  |  |  |  |  |

**3. Others**

• What workshop activities were most effective?

• What workshop activities do you think need to be improved?

• Overall opinion: If you attend a genetic nursing workshop in the near future, which area in genetic nursing would you like to pursue further?
